# Sex differences in patients with high risk HPV-associated and HPV negative oropharyngeal and oral cavity squamous cell carcinomas

**DOI:** 10.1186/s41199-018-0031-y

**Published:** 2018-06-20

**Authors:** Hong Li, Henry S. Park, Heather A. Osborn, Benjamin L. Judson

**Affiliations:** 1 Yale School of Medicine, New Haven, CT USA; 2 Department of Therapeutic Radiology, Yale New Haven Hospital, New Haven, CT USA; 3 Department of Surgery (Section of Otolaryngology), Yale New Haven Hospital, New Haven, CT USA; 4 Yale Cancer Center, 330 Cedar Street, PO Box 208062, New Haven, CT 06520-8062 USA

**Keywords:** Human papilloma virus, Sex difference, Head and neck cancer, Oropharyngeal cancer, Oral cavity cancer

## Abstract

**Background:**

Human papilloma virus (HPV)-associated head and neck cancer is now recognized as a distinct clinical entity from HPV-negative tumors, which are primarily associated with tobacco and alcohol exposure.Little is known, however, about the behavior of HPV-associated oropharynx (OP) and oral cavity (OC) SCCs as two distinct cancers and how sex affects the overall survival (OS) in these two cancers. The objective of our study is to determine if sex is associated with overall survival (OS) in patients with high-risk human papillomavirus (HPV)-positive and HPV-negative squamous cell carcinomas (SCC) in the oropharynx and oral cavity sites.

**Methods:**

This is a retrospective cohort study using a national database. Data were extracted from the National Cancer Database (NCDB) of patients diagnosed with OP or OC SCC from 2010 to 2014. Univariate and multivariate survival analyses were conducted with chi-square tests, Kaplan-Meier estimates, log-rank tests, and Cox proportional hazards multivariable modeling.

**Results:**

A total of 30,707 patients (13,694 OP HPV-associated, 7933 OP HPV-, 1220 OC HPV-associated, 7860 OC HPV-) were identified. In all four groups, women tended to be older and have lower T and N clinical classification than men. Though there were no significant differences in OS between the sexes in OP HPV-associated cancers, female sex was associated with worse OS in OP HPV- cancers (HR: 1.15; 95% CI 1.04–1.28, *p* = 0.004), whereas it was associated with improved OS in OC HPV-associated and HPV- cancers (HPV-associated: HR: 0.71; 95% CI 0.50–0.99, *p* = 0.048; HPV-: HR: 0.87; 95% CI 0.78–0.95, *p* = 0.004).

**Conclusion:**

The effect of sex on OS in OC and OP SCC appears to vary based on tumor location and HPV status. While the source of this difference in prognostic association is unclear, it may be related to an emerging difference in the biology of HPV carcinogenesis in these locations.

## Background

In the last 15 years, evidence has amassed on the human papilloma virus (HPV) as an important cause of head and neck squamous cell carcinomas (HNSCC). HPV-associated HNSCC is now recognized as a distinct clinical entity from HPV-negative HNSCC tumors [1], which are primarily associated with tobacco and alcohol exposure [2].

Given this recent discovery, many questions still remain regarding the epidemiology and management of patients with HPV-associated HNSCC. A subset of HNSCCs occurs in the oropharynx (OP). Chaturvedi and colleagues found that the incidence of OP cancers have been rising ~ 1–2% every year from 1973 to 2004 [3]. Despite HPV infection being common in both men and women, the incidence of HPV-associated OPSCCs is more than two-fold higher among men than women [4]. This sex-specific finding raises questions regarding possible differences in the biological presentation of the cancer between men and women.

OPSCCs are now hypothesized to behave distinctly compared to HNSCCs at other sites. HPV DNA has been discovered in tumors from other head and neck sites such as cancers of the oral cavity (OC) [5–7]. A recent study found that HPV-associated non-OPSCCs display a distinct immune microenvironment and clinical behavior compared to HPV-associated OPSCCs [8].

To date, few studies have alluded to the sex-related differences in the prognosis for OPSCCs and other HNSCCs. One retrospective, multi-institutional study [9] found sex to be a significant prognostic factor for overall survival (OS) in OPSCCs even after accounting for HPV status. Interestingly, the same study found that in non-OPSCCs, sex did not have any prognostic significance for OS.

Many studies in HPV-associated HNSCCs have examined all HNSCCs as a whole entity [10, 11]. Little is known, however, about the behavior of HPV associated OP and OC SCCs as two distinct cancers and how sex affects the OS in these two cancers. Therefore, this study aims to classify patient characteristics and investigate survival differences by sex in patients with HPV-associated and HPV- OPSCCs and OCSCCs.

## Methods

### Data

Data were extracted from the National Cancer Database (NCDB) from 2010 to 2014. The NCDB is a joint project of the Commission on Cancer and the American Cancer Society [12]. Cases are recorded from over 1500 accredited hospitals in the United States and Puerto Rico. The database represents over 70% of incident cases of cancer in the United States. Each hospital that participates in the registry is responsible for submitting and tracking patient and tumor level data on patients with malignant neoplastic diseases.

### Patient population

Our study population includes patients whose primary malignancy was diagnosed as squamous cell carcinoma of the oropharynx or oral cavity. The following *Internal Classification of Disease for Oncology, Third Edition* (ICD-O-3) histology codes were used for squamous cell carcinoma M8070–8073 and the following topography codes were used for oropharynx (OP): C09.0–09.1, C09.8–09.9 (tonsil) C10.0, C10.2–10.4 (other oropharynx) and C-01.9 (base of tongue), for oral cavity (OC) cancer C00.0–00.9 (lip), C02.0–02.4, C02.8–02.9 (other/unspecified parts of the tongue), C03.0–03.1, C03.9 (gum), C04.0–04.1, C04.8–04.9 (floor of mouth), C05.0–05.1, C05.8–05.9 (palate), C06.0–06.2, C06.8–06.9 (other/unspecified parts of the mouth).

HPV status was available for cases diagnosed 2010–2014 and was categorized as negative, positive for low-risk HPV types, positive for high-risk HPV types (HPV 16 and/or 18) and HPV status unknown. For our study, patients were classified as ‘HPV-positive’ if they tested positive for high-risk HPV types, and ‘HPV-negative’ if they received a negative HPV test. Patients with low-risk HPV types or unknown HPV status were excluded.

We examined patient demographic and tumor data (age at diagnosis, race, Charlson/Deyo score, primary tumor site, American Joint Commission on Cancer T and N classification, lymph node metastasis, primary treatment type, insurance status, median income quartiles, treatment facility type and location, and rural/urban classification of patient’s primary country of residence). Patients were excluded if they were younger than 18 years old, if TNM classification or primary treatment type was unknown. Primary treatment type was classified into the following groups: no treatment, radiation only, chemoradiation therapy, surgery and radiation, surgery and chemoradiation, surgery only and chemotherapy only.

### Statistical analysis

Data analyses were performed using SPSS 19.0 (IBM Corp., Armonk, NY). The comparison of mean age at diagnosis was analyzed using the Student’s t-test. Proportional distribution of race, Charleson/Deyo score, primary tumor site, T and N classification, lymph node metastasis, primary treatment type, insurance status, median income quartiles, treatment facility type and location, and rural/urban classification of patient’s primary country of residence were compared using chi-squared tests. Survival analysis was performed using Kaplan-Meier analysis. The comparison of survival rates among the groups was performed using the two-tailed log-rank test. The average follow up time for survival analysis in the dataset was 31.7 months. Cox proportional hazards regression model was used for multivariable survival analysis. Age, sex, race, Charleson/Deyo score (for OPSCCs only), T and N classification, site of primary tumor (for OPSCCs only), primary treatment type, insurance status and median income were entered a priori into the model. A two-sided *p*-value < 0.05 was considered statistically significant.

Our study is exempt from review by the Yale Human Research Protection Program because it uses a pre-existing, de-identified public database.

## Results

**Fig. 1:**
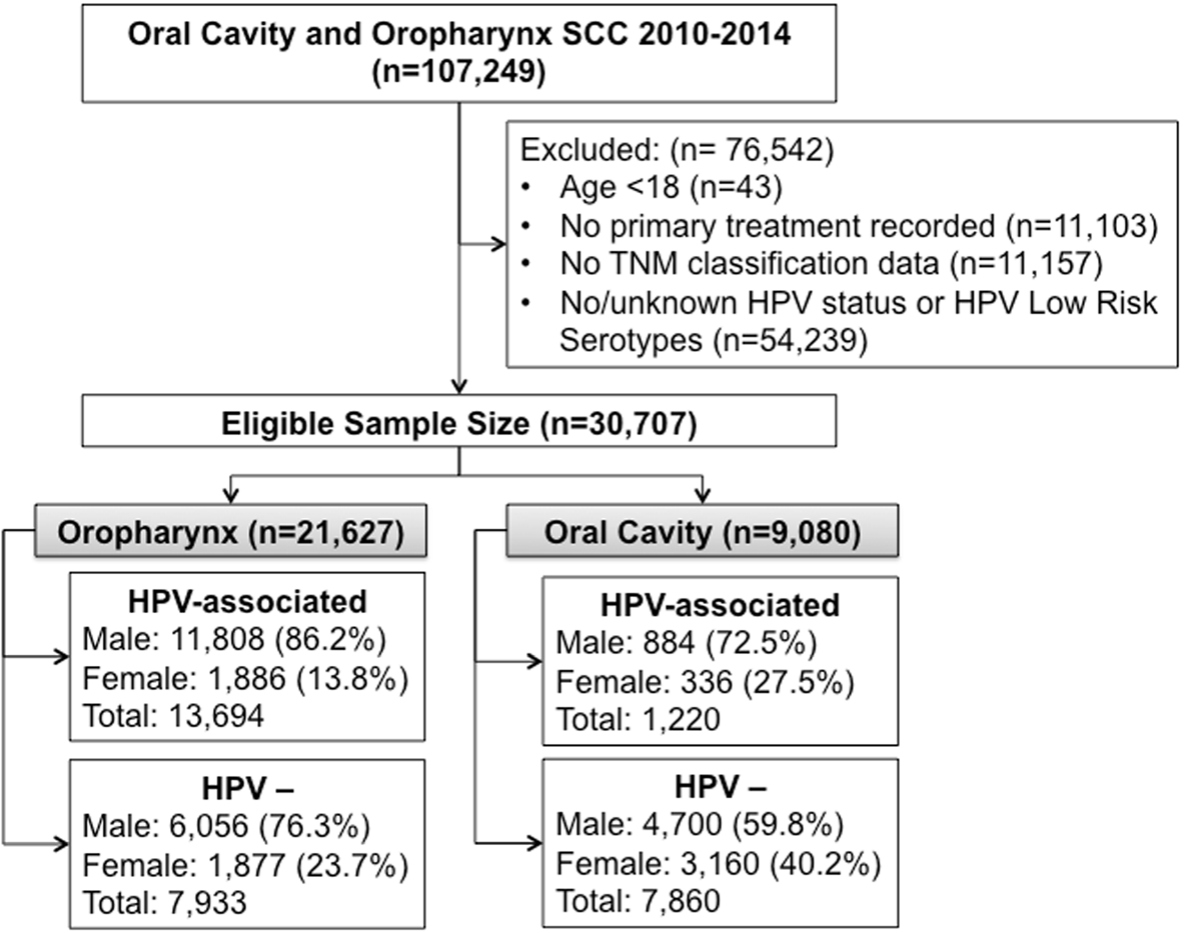
CONSORT diagram of total study population (*n* = 30,707)

Our study population (*n* = 30,707) included 13,694 OP HPV-associated; 7933 OP HPV- cancers; 1220 OC HPV-associated and 7860 OC HPV- cancers (Fig. 1). The presence of HPV was correlated with higher proportion of disease burden among men. Among the OP HPV-associated and HPV- cohorts, 86.2 and 76.3% of patients were men respectively. Among the OC HPV-associated and HPV- cohorts, 76.3 and 59.8% were men respectively. Each group was further analyzed for baseline characteristic differences by sex (Tables 1 and 2).

**Table 1:** Patient characteristics among those with oropharyngeal squamous cell carcinoma based on sex and HPV status

	Oropharynx HPV-associated					Oropharynx HPV -				
	Male		Female			Male		Female		
	Count	%	Count	%	*p*-value	Count	%	Count	%	*p*-value
Mean age (years)	58,69		59,65		< 0.001	60,74		61,66		< 0.001
Ethnicity					0.006					0.32
White	10,997	94.0%	1717	91.9%		5167	86.0%	1566	84.2%	
Black	538	4.6%	107	5.7%		709	11.8%	243	13.1%	
American Indian/Eskimo	22	0.2%	5	0.3%		13	0.2%	7	0.4%	
Asian/Pacific Islander	103	0.9%	28	1.5%		87	1.4%	32	1.7%	
Other	45	0.4%	12	0.6%		32	0.5%	11	0.6%	
Charlson/Deyo Score					< 0.001					0.72
0	9957	84.3%	1530	81.1%		4817	79.5%	1477	78.7%	
1	1486	12.6%	272	14.4%		942	15.6%	304	16.2%	
2	365	3.1%	84	4.5%		297	4.9%	96	5.1%	
AJCC Clinical Staging										
T Staging					< 0.001					0.002
T0	85	0.7%	13	0.7%		16	0.3%	6	0.3%	
T1	3225	27.4%	582	31.1%		1221	20.3%	428	23.0%	
T2	4834	41.1%	770	41.2%		2097	34.9%	671	36.0%	
T3	1925	16.4%	254	13.6%		1319	22.0%	339	18.2%	
T4	1354	11.5%	190	10.2%		1180	19.7%	380	20.4%	
TX	326	2.8%	61	3.3%		170	2.8%	38	2.0%	
N Staging					< 0.001					< 0.001
N0	1336	11.3%	318	16.9%		1410	23.4%	594	31.7%	
N1	1874	15.9%	424	22.5%		997	16.5%	344	18.3%	
N2	8023	68.1%	1088	57.8%		3298	54.6%	877	46.8%	
N3	522	4.4%	49	2.6%		293	4.9%	50	2.7%	
NX	33	0.3%	3	0.2%		37	0.6%	10	0.5%	
M Staging					0.812					0.013
M0	11,042	97.7%	1763	98.0%		5435	95.4%	1718	96.7%	
M1	265	2.3%	36	2.0%		264	4.6%	58	3.3%	
Primary Site					< 0.001					0.005
Base of Tongue	4845	41.0%	611	32.4%		2543	42.0%	745	39.7%	
Tonsil	6258	53.0%	1150	61.0%		2615	43.2%	796	42.4%	
Other OP	705	6.0%	125	6.6%		898	14.8%	336	17.9%	
Insurance Status					< 0.001					< 0.001
Not Insured	437	3.7%	71	3.8%		383	6.4%	111	6.0%	
Private Insurance/Managed Care	7264	62.2%	989	52.9%		2601	43.8%	732	39.5%	
Medicaid	753	6.4%	162	8.7%		703	11.8%	240	13.0%	
Medicare	2940	25.2%	634	33.9%		2121	35.7%	750	40.5%	
Other Government	290	2.5%	12	0.6%		132	2.2%	18	1.0%	
Median Income Quartiles 2008–2012					0.002					0.043
< $38,000	1472	12.5%	274	14.6%		1184	19.7%	389	20.9%	
$38,000–$47,999	2443	20.8%	435	23.1%		1335	22.2%	451	24.2%	
$48,000–$62,999	3265	27.7%	488	25.9%		1606	26.7%	491	26.4%	
$63,000 +	4588	39.0%	685	36.4%		1900	31.5%	530	28.5%	
Urban/Rural 2013					0.190					0.27
Metro	9826	85.3%	1573	84.8%		5053	85.5%	1539	84.0%	
Urban	1488	12.9%	259	14.0%		775	13.1%	265	14.5%	
Rural	200	1.7%	24	1.3%		80	1.4%	28	1.5%	
Facility Type					0.214					0.346
Community Cancer Program	743	6.4%	128	7.0%		531	8.9%	159	8.6%	
Comprehensive Community Cancer Program	3719	31.8%	596	32.7%		2153	36.0%	632	34.3%	
Academic/Research Program	5810	49.7%	907	49.8%		2618	43.8%	851	46.2%	
Integrated Network Cancer Program	1407	12.0%	192	10.5%		671	11.2%	199	10.8%	
Other specified types of cancer programs	0	0.0%	0	0.0%		0	0.0%	0	0.0%	
Facility Location					0.110					0.130
East	2440	20.9%	419	23.0%		1223	20.5%	398	21.6%	
South	3915	33.5%	610	33.5%		2454	41.1%	704	38.2%	
Midwest	3196	27.4%	493	27.0%		1408	23.6%	467	25.4%	
West	2128	18.2%	301	16.5%		888	14.9%	272	14.8%	
Treatment Group					< 0.001					< 0.001
No treatment	210	1.8%	31	1.6%		286	4.7%	90	4.8%	
Radiation only	868	7.4%	158	8.4%		508	8.4%	186	9.9%	
Radiation and Chemo	7185	60.8%	1011	53.6%		3571	59.0%	1004	53.5%	
Surgery and Radiation	726	6.1%	155	8.2%		240	4.0%	94	5.0%	
Surgery, Chemotherapy and Radiation	2027	17.2%	341	18.1%		725	12.0%	210	11.2%	
Surgery only	572	4.8%	155	8.2%		464	7.7%	218	11.6%	
Chemotherapy Only	220	1.9%	35	1.9%		262	4.3%	75	4.0%

**Table 2:** Patient characteristics among those with oral cavity squamous cell carcinoma based on sex and HPV status

	Oral Cavity HPV-associated					Oral Cavity HPV -				
	Male		Female			Male		Female		
	Count	%	Count	%	*p*-value	Count	%	Count	%	*p*-value
Mean age (years)	58,85		59,72		< 0.001	61,35		63,95		< 0.001
Ethnicity					0.502					0.512
White	804	91.6%	296	88.9%		4093	87.7%	2777	88.7%	
Black	44	5.0%	25	7.5%		371	7.9%	218	7.0%	
American Indian/Eskimo	2	0.2%	1	0.3%		15	0.3%	7	0.2%	
Asian/Pacific Islander	20	2.3%	9	2.7%		151	3.2%	103	3.3%	
Other	8	0.9%	2	0.6%		39	0.8%	26	0.8%	
Charlson/Deyo Score					0.11					0.92
0	689	77.9%	259	77.1%		3607	76.7%	2434	77.0%	
1	165	18.7%	57	17.0%		837	17.8%	552	17.5%	
2	30	3.4%	20	6.0%		256	5.4%	174	5.5%	
AJCC Clinical Staging										
T Staging					0.005					< 0.001
T0	4	0.5%	0	0.0%		8	0.2%	5	0.2%	
T1	251	29.3%	129	40.1%		1579	34.6%	1292	42.3%	
T2	277	32.4%	101	31.4%		1409	30.8%	914	29.9%	
T3	96	11.2%	32	9.9%		506	11.1%	271	8.9%	
T4	216	25.2%	56	17.4%		1039	22.7%	551	18.1%	
TX	12	1.4%	4	1.2%		29	0.6%	19	0.6%	
N Staging					0.003					< 0.001
N0	420	47.5%	198	59.5%		3001	64.0%	2226	70.7%	
N1	130	14.7%	46	13.8%		552	11.8%	332	10.5%	
N2	313	35.4%	86	25.8%		1038	22.2%	551	17.5%	
N3	15	1.7%	2	0.6%		57	1.2%	14	0.4%	
NX	6	0.7%	1	0.3%		38	0.8%	27	0.9%	
M Staging					0.939					0.004
M0	833	97.9%	313	97.8%		4339	97.7%	2959	98.6%	
M1	18	2.1%	7	2.2%		102	2.3%	41	1.4%	
Insurance Status					0.09					< 0.001
Not Insured	51	5.8%	23	6.9%		236	5.1%	132	4.2%	
Private Insurance/Managed Care	420	48.1%	145	43.5%		1935	41.9%	1197	38.5%	
Medicaid	90	10.3%	30	9.0%		515	11.1%	260	8.4%	
Medicare	286	32.8%	131	39.3%		1850	40.0%	1480	47.6%	
Other Government	26	3.0%	4	1.2%		85	1.8%	38	1.2%	
Median Income Quartiles 2008–2012					0.22					0.010
< $38,000	137	15.6%	47	14.0%		843	18.0%	512	16.3%	
$38,000–$47,999	206	23.4%	93	27.8%		1171	25.0%	725	23.0%	
$48,000–$62,999	264	30.0%	85	25.4%		1250	26.7%	863	27.4%	
$63,000 +	272	30.9%	110	32.8%		1426	30.4%	1046	33.2%	
Urban/Rural 2013					0.510					0.072
Metro	741	85.2%	287	87.8%		3799	82.8%	2579	83.9%	
Urban	114	13.1%	35	10.7%		708	15.4%	461	15.0%	
Rural	15	1.7%	5	1.5%		81	1.8%	35	1.1%	
Facility Type					0.507					0.967
Community Cancer Program	64	7.6%	20	6.6%		274	6.1%	188	6.3%	
Comprehensive Community Cancer Program	239	28.3%	75	24.8%		1226	27.3%	823	27.6%	
Academic/Research Program	466	55.2%	176	58.1%		2487	55.3%	1643	55.0%	
Integrated Network Cancer Program	75	8.9%	32	10.6%		507	11.3%	331	11.1%	
Other specified types of cancer programs	0	0.0%	0	0.0%		0	0.0%	0	0.0%	
Facility Location					0.990					0.026
East	178	21.1%	64	21.1%		995	22.1%	662	22.2%	
South	278	32.9%	98	32.3%		1660	36.9%	1014	34.0%	
Midwest	240	28.4%	87	28.7%		1149	25.6%	793	26.6%	
West	148	17.5%	54	17.8%		690	15.4%	516	17.3%	
Treatment Group					< 0.001					< 0.001
No treatment	29	3.3%	11	3.3%		163	3.5%	120	3.8%	
Radiation only	56	6.3%	26	7.7%		282	6.0%	217	6.9%	
Radiation and Chemo	269	30.4%	62	18.5%		717	15.3%	356	11.3%	
Surgery and Radiation	100	11.3%	37	11.0%		573	12.2%	396	12.5%	
Surgery, Chemotherapy and Radiation	146	16.5%	55	16.4%		806	17.1%	408	12.9%	
Surgery only	264	29.9%	139	41.4%		2072	44.1%	1620	51.3%	
Chemotherapy Only	20	2.3%	6	1.8%		87	1.9%	43	1.4%	

### Baseline characteristic differences

Within all four groups, women were on average older at age of diagnosis (*p* < 0.001 for each group). Women were generally diagnosed with cancers in earlier T and N clinical classification than men. In OP, this difference was most pronounced in N classification; in OP HPV-associated cancers, 39.4% women vs. 27.2% men had N0–1 cancers (*p* < 0.001), in OP HPV- cancers, 50.0% women vs. 39.9% men had N0–1 cancers (*p* < 0.001). In OC HPV-associated cancers, 40.1% women had T0–1 cancers vs. 29.8% men and in OC HPV- cancers (*p* = 0.005), 42.3% vs. 34.8% in women and men respectively (*p* < 0.001). Women in all four groups were more likely to be treated with a modality including surgery (surgery only, surgery and radiation, or surgery and chemo-radiation; p < 0.001 in each group). For insurance coverage, more women were covered by Medicare than men across all four study populations.

### Factors associated with survival in OPSCCs

**Fig. 2:**
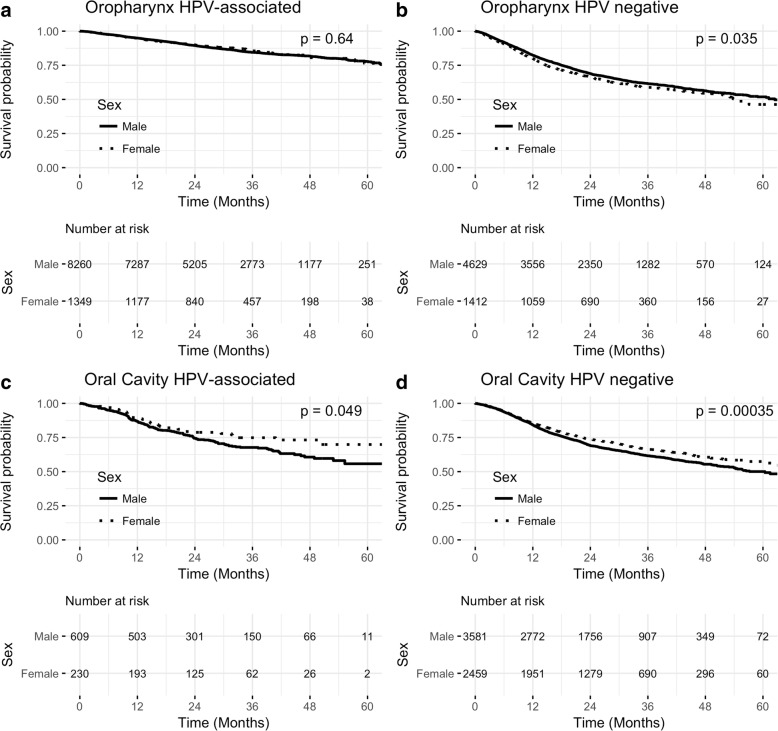
Kaplan-Meier survival and number at risk **a** OP HPV-associated: *p* = 0.638, **b** OP HPV negative: *p* = 0.035, **c** OC HPV-associated: *p* = 0.049, **d** OC HPV negative: *p* < 0.001

Kaplan-Meier survival analysis showed no difference in OS between the two sexes in OP HPV-associated cancers (*p* = 0.64; Figs. 2a). On multivariate analysis, after accounting for age at diagnosis, ethnicity, clinical T and N classification, primary disease site, primary treatment, insurance status and median income, female sex (HR: 0.93; 95% CI 0.79–1.009, *p* = 0.412) did not prove to be an independent prognostic factor for OS.

In OP HPV- cancers, men had a statistically significant better OS than women on Kaplan Meier survival analysis (*p* = 0.035, Fig. 2b). In multivariate analysis, female sex (HR: 1.15; 95% CI 1.04–1.28, *p* = 0.004) continued to be an independent prognostic factor for worse OS in OP HPV- cancers even after controlling for other variables (as described previously, Table 3).

**Table 3:** Cox proportional hazards regression analysis for patients with oropharyngeal squamous cell carcinoma

	Oropharynx HPV-associated		Oropharynx HPV -	
	HR (95% CI)	*P*	HR (95% CI)	*P*
Mean age	1.02 (1.01–1.03)	< 0.001	1.01 (1.00–1.02)	< 0.001
Ethnicity				
White	1.00		1.00	
Black	0.86 (0.67–1.10)	0.25	1.14 (1.01–1.30)	0.03
American Indian/Eskimo	0.61 (0.15–2.47)	0.49	0.22 (0.03–1.58)	0.13
Asian/Pacific Islander	0.52 (0.24–1.10)	0.09	0.75 (0.50–1.14)	0.19
Other	0.63 (0.15–2.52)	0.51	1.29 (0.67–2.49)	0.44
**Sex**				
**Men**	**1.00**		**1.00**	
**Women**	**0.93 (0.79–1.09)**	**0.412**	**1.15 (1.04–1.28)**	**0.004**
Charlson/Deyo Score				
0	1.0		1.0	
1	1.42 (1.23–1.65)	< 0.001	1.31 (1.17–1.46)	< 0.001
2	1.97 (1.56–2.48)	< 0.001	1.49 (1.25–1.77)	< 0.001
AJCC Clinical Staging				
T Staging				
T0	1.00		1.00	
T1	2.61 (0.64–10.5)	0.18	1.31 (0.32–5.30)	0.70
T2	4.24 (1.05–17.0)	0.04	1.93 (0.48–7.79)	0.35
T3	6.47 (1.60–26.1)	0.01	3.26 (0.81–13.1)	0.10
T4	9.92 (2.45–40.0)	0.00	4.35 (1.08–17.5)	0.04
TX	3.93 (0.93–16.4)	0.06	2.65 (0.64–10.9)	0.18
N Staging				
N0	1.00		1.00	
N1	0.81 (0.64–1.01)	0.07	0.95 (0.82–1.10)	0.53
N2	1.14 (0.95–1.37)	0.14	1.10 (0.98–1.24)	0.09
N3	2.06 (1.58–2.67)	< 0.001	1.76 (1.45–2.15)	< 0.001
NX	0.73 (0.29–1.83)	0.51	1.47 (0.91–2.36)	0.11
Primary Site				
Base of Tongue	1.00		1.00	
Tonsil	1.03 (0.91–1.16)	0.63	0.87 (0.79–0.96)	0.01
Other OP	1.48 (1.21–1.81)	< 0.001	1.15 (1.02–1.30)	0.02
Insurance Status				
Not Insured	1.00		1.00	
Private Insurance/Managed Care	0.53 (0.41–0.68)	< 0.001	0.61 (0.51–0.72)	< 0.001
Medicaid	1.04 (0.78–1.38)	0.77	1.11 (0.93–1.34)	0.23
Medicare	0.99 (0.76–1.30)	0.98	0.94 (0.78–1.13)	0.55
Other Government	0.96 (0.63–1.46)	0.85	0.96 (0.67–1.36)	0.83
Median Income Quartiles 2008–2012				
< $38,000	1.00		1.00	
$38,000–$47,999	0.89 (0.75–1.06)	0.21	0.9 (0.79–1.02)	0.11
$48,000–$62,999	0.78 (0.65–0.93)	0.01	0.83 (0.73–0.94)	0.01
$63,000 +	0.65 (0.54–0.77)	< 0.001	0.73 (0.64–0.83)	< 0.001
Treatment Group				
No treatment	1.00		1.00	
Radiation only	0.34 (0.24–0.48)	< 0.001	0.44 (0.35–0.54)	< 0.001
Radiation and Chemo	0.22 (0.16–0.29)	< 0.001	0.27 (0.23–0.33)	< 0.001
Surgery and Radiation	0.16 (0.10–0.24)	< 0.001	0.20 (0.14–0.28)	< 0.001
Surgery, Chemotherapy and Radiation	0.21 (0.15–0.29)	< 0.001	0.29 (0.23–0.35)	< 0.001
Surgery only	0.21 (0.14–0.32)	< 0.001	0.37 (0.29–0.47)	< 0.001
Chemotherapy Only	1.08 (0.76–1.54)	0.64	0.94 (0.76–1.18)	0.64

The hazard of death was notably higher for both OP HPV-associated and HPV- cohorts with increasing age, higher T and N classification, cancers at sites other than base of tongue or tonsils and patients with no primary treatment (Table 3).

### Factors associated with survival in OCSCCs

Kaplan-Meier survival analysis showed that among OC cancers, women had better OS than men in both HPV-associated and HPV- cancers (*p* = 0.049, *p* < 0.001 respectively, Fig. 2c, d).

In contrast to the varying prognostic roles of female sex in OPSCCs, in OCSCCs, female sex remained a strong prognostic factor for better OS in both HPV-associated and HPV- cancers (HPV-associated: HR: 0.71; 95% CI 0.0.50–0.99, *p* = 0.048; HPV-: HR: 0.87; 95% CI 0.78–0.95, *p* = 0.004; Table 4) after controlling for over variables. In OC HPV-associated cancers, age (HR: 1.02; 95% CI 1.00–1.04, *p* = 0.01) and black race (HR: 1.88; 95% CI 1.14–3.11, *p* = 0.013) were significant predictors of OS in patients. In OC HPV- cancers, age (HR: 1.02; 95% CI 1.02–1.02, *p* < 0.001), N classification (*p* < 0.001) and having higher median income $63,000+ ((HR: 0.77; 95% CI 0.67–0.88, *p* < 0.001), and having treatment (over no treatment; *p* < 0.001 for all except chemotherapy only group *p* = 0.31) were all significant predictors of OS.

**Table 4:** Cox proportional hazards regression analysis for patients with oral cavity squamous cell carcinoma

	Oral Cavity HPV-associated		Oral Cavity HPV -	
	HR (95% CI)	*P*	HR (95% CI)	*P*
Mean age	1.02 (1.00–1.04)	0.010	1.02 (1.02–1.02)	< 0.001
Ethnicity				
White	1.00		1.00	
Black	1.88 (1.14–3.11)	0.013	0.93 (0.79–1.09)	0.41
American Indian/Eskimo	a		1.18 (0.52–2.64)	0.68
Asian/Pacific Islander	1.60 (0.64–4.00)	0.312	0.93 (0.71–1.22)	0.63
Other	0.65 (0.09–4.78)	0.678	0.54 (0.30–0.99)	0.05
**Sex**				
**Men**	**1.00**		**1.00**	
**Women**	**0.71 (0.50–0.99)**	**0.048**	**0.87 (0.78–0.95)**	**0.004**
AJCC Clinical Staging				
T Staging				
T0	1.00		1.00	
T1	0.36 (0.07–1.65)	0.189	0.45 (0.14–1.43)	0.18
T2	0.61 (0.13–2.80)	0.529	0.79 (0.25–2.49)	0.70
T3	0.90 (0.19–4.21)	0.903	1.07 (0.34–3.38)	0.90
T4	1.33 (0.29–5.98)	0.707	1.19 (0.37–3.73)	0.76
TX	0.67 (0.11–4.00)	0.666	1.36 (0.39–4.70)	0.63
N Staging				
N0	1.00		1.00	
N1	1.07 (0.70–1.63)	0.743	1.54 (1.34–1.77)	< 0.001
N2	1.08 (0.75–1.55)	0.651	1.72 (1.52–1.94)	< 0.001
N3	0.88 (0.26–2.93)	0.836	2.12 (1.49–3.03)	< 0.001
NX	1.30 (0.17–9.88)	0.795	1.19 (0.75–1.89)	0.45
Insurance Status				
Not Insured	1.00		1.00	
Private Insurance/Managed Care	0.74 (0.42–1.32)	0.319	0.81 (0.65–1.00)	0.06
Medicaid	1.82 (0.96–3.43)	0.064	1.27 (1.00–1.60)	0.043
Medicare	1.32 (0.72–2.41)	0.355	1.04 (0.83–1.30)	0.72
Other Government	0.83 (0.31–2.19)	0.713	1.17 (0.78–1.76)	0.44
Median Income Quartiles 2008–2012				
< $38,000	1.00		1.00	
$38,000–$47,999	1.38 (0.88–2.17)	0.160	0.84 (0.73–0.96)	0.02
$48,000–$62,999	1.49 (0.96–2.33)	0.075	0.90 (0.79–1.03)	0.14
$63,000 +	1.37 (0.87–2.16)	0.169	0.77 (0.67–0.88)	< 0.001
Treatment Group				
No treatment	1.00		1.00	
Radiation only	0.56 (0.25–1.23)	0.151	0.49 (0.37–0.63)	< 0.001
Radiation and Chemo	0.42 (0.22–0.82)	0.011	0.44 (0.35–0.55)	< 0.001
Surgery and Radiation	0.23 (0.10–0.50)	< 0.001	0.33 (0.26–0.43)	< 0.001
Surgery, Chemotherapy and Radiation	0.59 (0.30–1.15)	0.127	0.42 (0.33–0.53)	< 0.001
Surgery only	0.48 (0.24–0.94)	0.033	0.37 (0.29–0.45)	< 0.001
Chemotherapy Only	1.18 (0.49–2.82)	0.710	0.84 (0.61–1.16)	0.31

^a^Insufficient sample size

## Discussion

HPV status and its importance as a prognostic marker in oropharyngeal SCCs has been well established [13, 14]. The prognostic associations of HPV status with other clinical factors such as sex and primary tumor location have not been well investigated. Given that HNSCCs affect the two sexes disproportionately (80% men), we hypothesized that sex will be a prognostic factor for survival in HNSCCs. Our study found that sex does appear to play a distinct role in predicting OS and that the prognostic value of sex is dependent on HPV status and location of primary tumor. This finding is consistent with the idea that HPV-driven cancers in non-OP locations exhibit distinct clinical behavior and possess unique risk factors than HPV-driven cancers in OP [8, 9].

Molecular underpinnings of the HPV infection between the two sexes also vary. One Finnish study examining the clearance of HPV DNA using oral rinses between spouses found earlier virus clearance in men than in women as well as significantly different cumulative clearance rates (5% vs. 0% clearance in men and women respectively over 24 months) [15]. In a long-term prospective 6 year study of asymptomatic HPV infections, Syrjänen and colleagues found a 5.5 fold number of viral HPV copies in women than in men who were able to clear the infection [16]. Although similar copy numbers were found between sexes for those with persistent infections, 71% of the HPV DNA was integrated or mixed in women vs. 57% in men. Full integration of the HPV episome into human chromosomes has been shown to be an early event in cervical carcinogenesis [17, 18], though its role in oral mucosal carcinogenesis is still debated. Nonetheless, these studies reflect a distinction in HPV’s molecular behavior between sexes that needs to be further categorized.

Prior studies have been inconclusive on the significance of sex as a prognostic marker for overall survival. A recent two-institution retrospective study found sex to be prognostic in OPSCCs even after accounting for HPV-status [9]. The authors examined 860 patients with OPSCCs (including HPV-associated and HPV- patients) and performed a multivariate regression model. Our study utilizes more targeted patient subgroups that specifically examines the role of sex among HPV-associated or HPV- patients. To our knowledge, our study is the largest study with patients and their HPV status spanning across the entire U.S. As a result, our sample provides the power for the subgroup analyses for the detection of differences in sex. However, due to the nature of the national cancer registry, there is inherent uncertainty to the nature of our data as the quality of the data relies on the accuracy of data entry, diagnosis and treatment at over 1500 hospitals. In comparison, Fakhry et al.’s two-institution study limits their data inaccuracies due to a smaller sample size.

Existing research has shown that women have a significant survival benefit in many cancers outside of the head and neck region [19]. However, for HPV- OPSCCs, we found the opposite where men have better survival than women. This similar trend also exists in patients with bladder cancer [20, 21]. The reason for this observed survival advantage is unknown. Preclinical studies support a role for sex hormones as cofactors for HPV-related malignancies [22, 23] though other unidentified factors may also be responsible for this unique sex-specific finding. One study found the progesterone antagonists and nuclease-resistant oligomers containing HPV-16 response element are able to abrogate cell growth and E6/E7 gene transcription [22]. Another study examining HPV-induced laryngeal tumors found estradiol stimulated proliferation while 2-hydroxyestrone was anti-proliferative [23]. Both preclinical studies found hormonal interactions using HPV-associated tumor models, thus this does not fully explain our findings in the HPV- OPSCC cohort. Perhaps there exists an interaction between HPV and sex hormones in the OP sub-site, which improves the survival of women thus equalizing overall survival between the two sexes. Nonetheless, we acknowledge the proximity of the Kaplan-Meier survival curve between the two sexes in the HPV- OPSCC cohort. Given the absence of tobacco and alcohol data, it is possible that the two sexes may have no survival difference in HPV- OPSCCs.

Interestingly, in our OCSCC study population, women were shown to have better survival than men in both the HPV-associated and HPV- group. This finding contrasts with the role that sex plays in OPSCCs and is consistent with the developing hypothesis that OP and non-OP SCCs are distinct cancers. Risk factors for OCSCC are well established: alcohol, tobacco and betel nut chewing [2, 24]. Current rates of tobacco usage in the US are lower in women than in men [25]. As a result, a lower overall lifetime exposure to tobacco may partly explain the survival advantage among women in OCSCC. There is a new growing body of research interested in characterizing HPV in non-OP sites. A molecular study of 520 HNSCCs profiling the gene-expression signature of HPV-associated OP and non-OP sites found there to be two distinct tumor immune microenvironments [8].

While our study did not directly test for the role of HPV within the OCSCC group, the similarity in risk factors between the HPV-associated and HPV- OCSCC groups infers that HPV may only play a minor prognostic role in OC cancers. A recent study by our group [26] found HPV to be associated with improved survival at the OCSCC subsite, though the survival advantage noted at the oral cavity subsite was not as great as that at the oropharynx subsite.

In our study, we found women were generally diagnosed with earlier T and N staged cancers than men. Earlier detection of cancers would lead to better prognosis [27]. From a health behavior perspective, this finding may be explained by the consistent underutilization of preventative healthcare by men leading to a delay in early diagnosis [28, 29]. It has been hypothesized that women have more frequent contact with healthcare professionals due to pregnancy, childcare and hormone replacement therapy as well as women having more interest in health [28, 30].

The NCDB database, as a source, has well-documented limitations [31]. We were unable to account for every variable that may influence survival (e.g. alcohol, tobacco use, and other comorbidities), as these data were not captured by NCDB. In addition, the database does not capture other causes of OC and OP cancers that may influence survival. Specifically, studies have shown that patients with cancer from previous leukoplakia [32] or oral mucositis [33] leading to earlier cancer detection is associated with improved survival, where as patients with cancer from immunosuppression [34] tend to have worse survival. The type of testing (PCR, ISH for HPV DNA vs. p16) for HPV status may vary depending on each institution and reporting agency. Furthermore, the source of the sample may not necessarily derive from the primary site. There are likely low rates of misclassification due to the nature of the registry of the data; however, any misclassification is likely to have been evenly distributed across our four subgroups. Our retrospective study focuses on OS, not cancer-specific survival. The absence of cause-specific survival data in NCDB makes in plausible that other causes of death such as treatment derived toxicities, secondary primary cancer and comorbid cardiovascular, pulmonary and metabolic syndrome causes which are more prominent in men may contribute to the difference in mortality seen between the two sexes. In addition, other general cancer risk factors such as tobacco and alcohol as well as high-risk sex behavior associated with HPV+ transmission [35] may also influence the survival difference seen.

## Conclusion

In summary, the effect of sex on outcomes of OP and OC SCCs appears to vary based on primary tumor location and HPV status. Notably, sex does not appear to affect the prognosis of HPV-associated OPSCCs after accounting for other risk factors. Men with HPV- OPSCCs appear to have a better prognosis for survival than women, though women appear to have a better prognosis in OCSCCs regardless of HPV-status. Given these results, we recommend further studies to investigate the clinical behavior and the sex-specific pathophysiological biology of HPV-associated HNSCCs and explore opportunities to further eliminate disparities in our patients.

## Abbreviations

HNSCC: Head and neck squamous cell carcinomas
HPV: Human papilloma virus
NCDB: National Cancer Database
OC: Oral cavity
OP: Oropharynx
OS: Overall survival
SCC: Squamous cell carcinomas
